# Effectiveness of Supplemental Feeding Combined with Nutrition Education on Child Nutrition Outcomes in Kenya

**DOI:** 10.3390/nu18203327

**Published:** 2026-10-11

**Authors:** Elyvine Ingabire-Gasana, Duke Appiah, Surya Raj Niraula, Oak-Hee Park, Mary Murimi

**Affiliations:** 1 Department of Nutritional Sciences, Texas Tech University, Lubbock, TX 79409, USA; surya.niraula@ttu.edu (S.R.N.); oak-hee.park@ttu.edu (O.-H.P.); mary.murimi@ttu.edu (M.M.); 2 Julia Jones Matthews School of Population and Public Health, Texas Tech University Health Sciences Center, Lubbock, TX 79430, USA; duke.appiah@ttuhsc.edu

**Keywords:** nutrition education, complementary feeding, pastoralists, malnutrition

## Abstract

**Background/Objectives**: Nutrition education coupled with supplemental feeding has been shown to be an effective intervention to combat child undernutrition. However, evidence of such interventions remains scarce in pastoralist communities in Kenya despite a high burden of child undernutrition. This study aimed to assess the combined effect of a theory-based nutrition education intervention and consumption of Harvest Lentil Vegetable Blend on feeding practices and the nutritional status of Turkana children in Kenya. **Methods**: This study was a two-month-long quasi-experimental controlled trial, entailing children aged 6 months to 59 months (*n* = 318) and their caregivers (*n* = 284). Children in the intervention group received one daily meal of supplemental food for a total of eight weeks, and their caregivers received a theory-based nutrition education intervention. Linear mixed effects models and generalized estimating equations were employed to assess the combined effect of the interventions. **Results**: Compared to the control group, children in the intervention group were significantly more likely to consume animal-source foods, meet minimum dietary diversity, and consume more meals at the study endpoint. This study further observed a trend of greater improvement across key indicators of child nutrition status in the intervention group compared to the control group. However, none of the changes in children’s nutrition status were statistically significant. **Conclusions**: This study adds to the literature by demonstrating that an integrated nutrition intervention that coupled a theory-based nutrition education intervention with a blanket supplemental feeding program was effective in improving child-feeding practices in a resource-restrained, pastoralist community in Kenya.

## 1. Introduction

Child undernutrition continues to be a significant global health problem, with 23.2% of children below five years old affected by stunting, 6.6% affected by wasting, and 12.2% being underweight [1,2]. Africa carries a disproportionately heavier burden of malnourished children, accounting for 43% of the world’s stunted children and 27% of those affected by wasting [1]. More specifically, 30.3% of children below five years old living in Africa have stunted growth, 5.4% are affected by wasting, and 15.7% are underweight [1,3].

Child undernutrition is associated with various interconnected and multisectoral factors [4]. According to United Nations International Children’s Emergency Fund (UNICEF) determinants of childhood undernutrition can be classified as immediate, underlying, and enabling [4]. The immediate determinants of undernutrition entail inadequate food intake and infectious diseases, associated with inadequate nutrient intake and impaired nutrient uptake, respectively [4,5]. The underlying determinants are household- and community-level determinants that potentially lead to poor dietary intake and an increased burden to diseases [4,5,6]. These include factors like food insecurity; poor infant and young child-feeding (IYCF) practices; and inadequate water, hygiene, and sanitation (WASH) practices [4]. The enabling determinants associated with childhood undernutrition are the socio-economic, cultural and political factors, and man-made and natural crises, from which the underlying causes emerge [4]. Examples include low maternal education levels, poverty, harmful norms, poor nutrition governance, and political instability [6,7].

Given the complexity of childhood undernutrition, multisectoral approaches to its prevention and treatment have been enacted worldwide [8]. Nutrition education interventions that promote proper complementary feeding and WASH practices have been shown to be cost-effective in combating childhood undernutrition [9,10]. Research further shows that complementary feeding education interventions are most effective when combined with supplemental food provision in regions experiencing widespread food insecurity [9]. Such supplemental feeding programs can either be targeted only at malnourished children or implemented on a larger scale to include all children at risk [11].

Child undernutrition in Turkana County, a predominantly pastoralist county in Kenya, remains a public health issue with a prevalence much higher than the national average [12]. Despite significant economic growth in Kenya and Turkana County in the last eight years, childhood undernutrition in Turkana has not improved [12,13]. For example, in the 2014 Kenyan Demographic Health Survey (KDHS), the prevalence of stunting, wasting, and underweight in Turkana County was 23.9%, 22.9%, and 34%, respectively [13]. Based on the 2022 KDHS report, the prevalence has not decreased with 23%, 22.6%, and 31.7% of Turkana children still affected by stunting, wasting, and underweight, respectively [12]. Despite the high levels of child undernutrition in Turkana County, there is a dearth of research on the effectiveness of interventions in improving child nutrition status such as theory-based nutrition education interventions and supplemental feeding programs. The present study was designed to bridge this gap, with the aim of assessing the effectiveness of an integrated community-based nutrition intervention, combining both nutrition education and supplemental food provision, on caregivers’ child-feeding practices and mitigation of undernutrition among Turkana children below five years old.

## 2. Materials and Methods

### 2.1. Study Design and Setting

**Figure 1:**
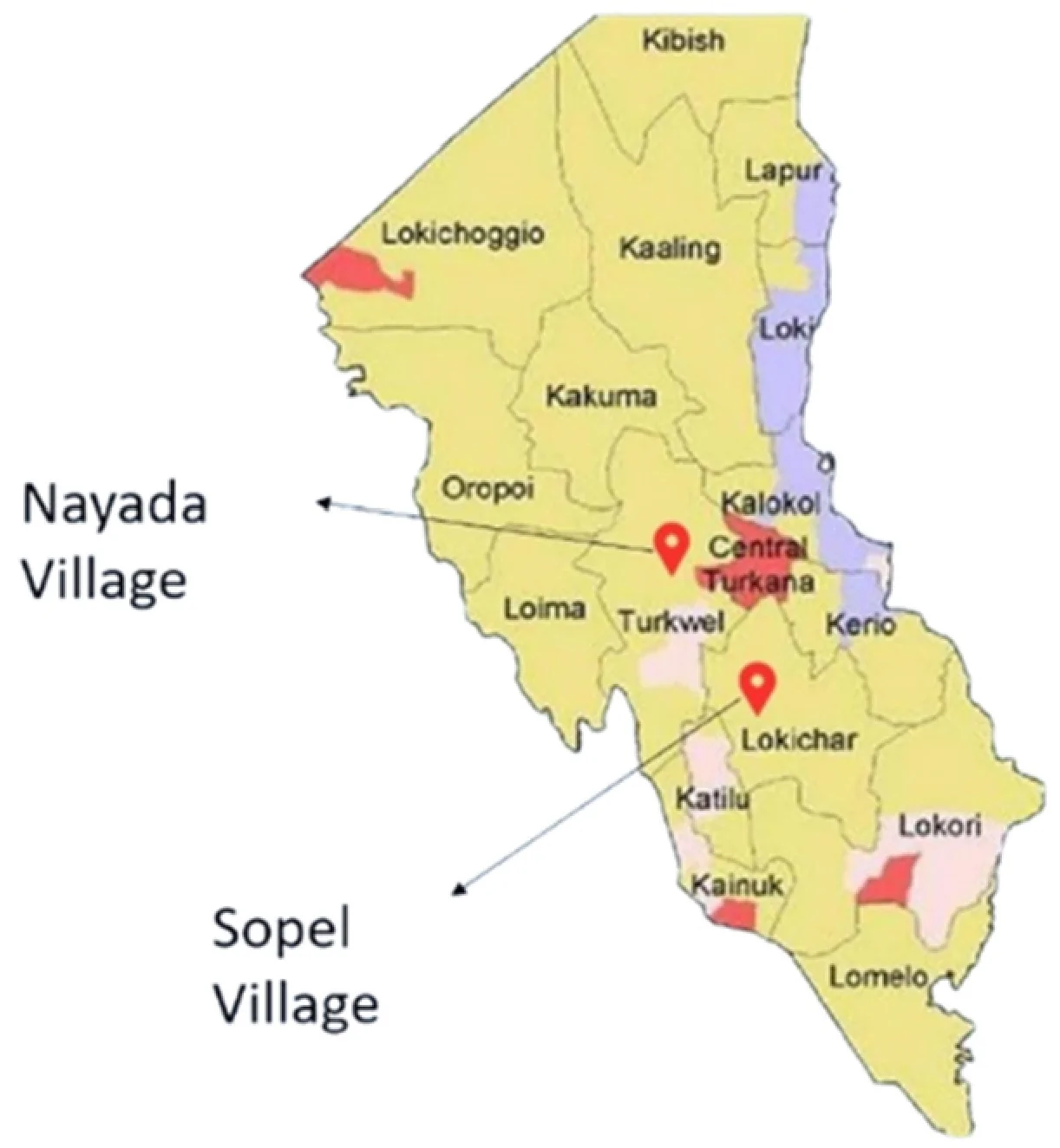
Study location.

This study was a two-month-long quasi-experimental controlled trial, implemented from June to August 2023 in Turkana County, Kenya. Specifically, this study was conducted in Nayada and Sopel villages located in Turkana Central and Turkana East, respectively (Figure 1). These two villages were purposively selected because they were similar in terms of socio-economic status, but far enough apart, as sub-locations in Turkana County (47 km apart), to minimize data contamination between the intervention and control groups. Non-random allocation to the intervention and control groups was conducted at the community level. Sopel village was designated as the intervention group, while Nayada was designated as the control group.

### 2.2. Study Participants and Recruitments

The target population of this study was children aged 6 months to 59 months and their caregivers. Recruitment of the participants was done in partnership with Share International Inc., a Turkana community-based non-governmental organization. This organization assisted in identifying two communities in which the study took place. The inclusion criteria for this study were: (i) children between 6 months and 59 months; (ii) children with no known allergic reactions to rice, lentils, carrots and onions; (iii) being a primary caregiver of a child aged 6 months to 59 months; (iv) being a permanent resident of Nayada and Sopel villages; and (v) willingness to participate in the study. Multiple children from the same family were recruited if they met the study inclusion criteria.

The sample size calculation was based on the difference in weight change as the study’s primary outcome. The expected difference was based on a weight gain of 0.47 ± 0.35 kg among children in the control group and 0.62 ± 0.47 kg among those receiving Harvest Lentil Vegetable Blend as found in a similar feeding study in rural Malawi [14]. The sample size calculation assuming 80% power, a 95% confidence level, and 20% attrition rate showed that a minimum of 144 child–caregiver pairs per group would be required for the current study.

### 2.3. Interventions

Children in the intervention group received a daily meal of Harvest Lentil Vegetable Blend (six days/week) for eight weeks, and their caregivers received sessions of nutrition education on IYCF practices and WASH practices. Children in the control group continued to receive routine growth monitoring by community health workers, with children identified as being at risk of or having malnutrition referred to the local clinic. As compensation for their time, participants in the control group were given one bag of supplemental food (1 kg) after each data collection session. Prior to the implementation of the intervention, children in both control and intervention groups received deworming medication to avoid confounding bias of intestinal infections. This activity was undertaken with the assistance of the local health clinic and community health workers.

#### 2.3.1. Consumption of Harvest Lentil Vegetable Blend

In this study we implemented a blanket supplemental feeding program where all the participating children in the intervention group received a meal of Harvest Lentil Vegetable Blend. This dehydrated supplemental food is produced from natural ingredients, namely rice, carrots, lentils and onions. This product was prepared by mixing it with water and boiling the mixture for approximately 25–35 min to a mid-consistency.

The feeding program was done on-site, with food portions allocated based on the child’s age as follows: (i) children aged 6 months to 12 months received three-quarters of a cup of the supplemental food (∼52 kcal/d, 15 g of dry weight), (ii) children aged 13 months to 23 months received 1 cup of the supplemental food (∼69 kcal/d, 20 g of dry weight), (iii) and children aged 24 months to 59 months received 2 cups of the supplemental food (∼138 kcal/d, 40 g of dry weight) [15]. The cooked food was provided 6 days a week (Monday through Saturday) for a total of eight weeks at the study site. The dosage and duration of the supplemental feeding were informed by a three-week pilot study conducted in Turkana, which found significant changes in nutrition outcomes among children aged 6–59 months who consumed Harvest Lentil Vegetable Blend, while their caregivers received a nutrition education intervention [15]. The nutrient composition of Harvest Lentil Vegetable Blend is shown in Appendix A Table A1.

#### 2.3.2. Nutrition Education Intervention

**Figure 2:**
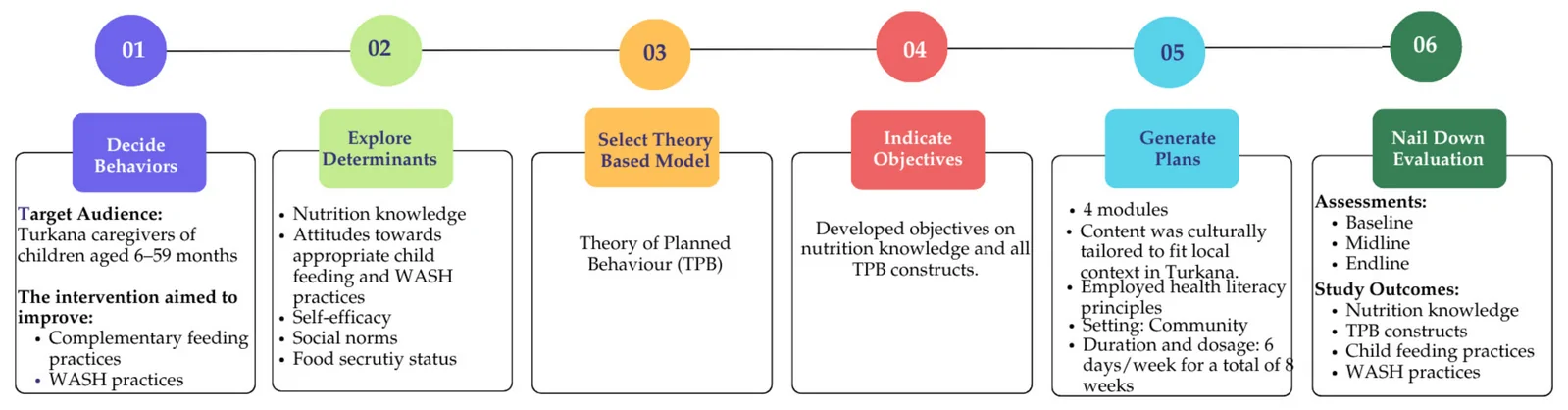
Overview of the DESIGN procedure applied in developing the nutrition education intervention.

The nutrition education intervention aimed to improve child-feeding practices and WASH practices among caregivers of children aged 6 months to 59 months residing in Turkana County, Kenya. The intervention followed the DESIGN procedure, a systematic six-step stepwise method used to design nutrition education interventions [16], and it was guided by formative assessments conducted in the summer of 2022 [15]. The six steps of the procedure, detailing how the intervention was designed, are described in Figure 2.

The development of the nutrition education modules was informed by the Theory of Planned Behavior (TPB), which postulates that an individual’s intentions to perform a behavior directly predict the behavior [17]. Individuals’ intentions are predicted by the interplay of three attributes: attitudes, subjective norms, and perceived behavior control [17]. This nutrition education intervention had four modules developed using evidence-based recommendations from the UNICEF community infant and young child feeding counseling package and from a previous pilot study conducted in Turkana County [15,18]. Module one covered the recommended breastfeeding practices, including early initiation of breastfeeding, exclusive breastfeeding, and continued breastfeeding. The second module was on young child-feeding practices, with a focus on the quality and quantity of food given to children and how to care for a sick child. The third module entailed WASH practices such as disinfection of drinking water, harms of open defecation, and other personal and household hygiene practices. The fourth module served as a comprehensive review of all previous modules, offering clarification on concepts that were not fully understood. Refer to Table 1 for more information on the content in each module.

The nutrition education sessions were held in the morning, Monday through Saturday, for a total of eight weeks in a community building, and each session lasted for approximately 60–75 min. The duration and intensity of the nutrition education intervention were informed by formative assessments. The nutrition education intervention was implemented by two community nutritionists, who both have many years of experience in nutrition education intervention, particularly in low-income countries. A literacy teacher, fluent in English and Kiturkana, with extensive experience in interpreting health-related interventions, served as an interpreter during the nutrition education sessions. To accommodate caregivers’ low literacy levels, we utilized multiple health literacy techniques such as using a local interpreter, brainstorming actionable goals, the teach-back technique, repeating the same message multiple times, and using clear visuals [19,20].

### 2.4. Assessment Tools and Data Collection

Data collection was conducted with the help of six trained data collectors fluent in both Kiturkana and English. The study questionnaire was translated into Kiturkana, the local language, by a professional translator and checked for accuracy. With the exception of sociodemographic characteristics, which were measured only at baseline, all other data were collected at baseline, midline and endline. At midline and endline assessments, data were collected on Monday, following a non-feeding day (Sunday).

#### 2.4.1. Demographic Information

The demographic information obtained included the caregiver’s age, level of education, occupation, and types and number of livestock owned. The remainder of the data collected included the child’s age, gender, and infectious disease occurrence in the previous 14 days.

#### 2.4.2. Anthropometric Measurements

The children’s anthropometric measurements collected in this study were weight and height. The child’s height was measured using a Shorr Board® measuring board (Weigh and Measure, LLC, Olney, MD, USA) and was recorded to the nearest 0.1 cm. Weight measurements were taken using an electronic weighing scale (ROTLIN) and recorded to the nearest 0.01 kg. The child Z-scores, weight-for-height (WHZ) and weight-for-age (WAZ), were computed using the World Health Organization (WHO) Anthro Survey Analyzer, version 3.2.2 [21].

#### 2.4.3. Child-Feeding Practices

Child-feeding practices were assessed using questions adapted from the Food and Agriculture Organization (FAO) guidelines for assessing nutrition-related knowledge, attitudes, and practices, WHO and UNICEF guidelines on the indicators to assess the IYCF practices, and from a 24 h food recall [22,23].

To assess children’s dietary diversity, caregivers were asked if their children consumed food from the following eight food groups: (i) breastmilk; (ii) grains, roots, and tubers; (iii) pulses, nuts, and seeds; (iv) dairy products; (v) flesh foods; (vi) eggs; (vii) vitamin-A-rich fruits and vegetables; and (viii) other fruits and vegetables [23]. A food group was assigned a score of one if a child consumed at least one food item from that group; otherwise, it was scored as zero [23]. To calculate the dietary diversity scores, all the food groups consumed were added up. A child was considered to have met the minimum dietary diversity (MDD) if they consumed at least five food groups for ages 6–23 months and at least four food groups for ages 24–59 months (excluding breastmilk) [23,24].

The proportion of children consuming animal-source foods (ASFs) was calculated by dividing the number of children who consumed at least one food item from the dairy products, flesh foods, and eggs food groups by the total number of children in the sample. The meal frequency was calculated by summing all the feedings a child partook in throughout the day, including solid, semi-solid or soft foods, as well as milk feed for non-breastfed children [23].

#### 2.4.4. Household Food Security Status

The prevalence of food insecurity among participants was assessed using the Household Food Insecurity Access Scale (HFIAS) [25]. This questionnaire has nine occurrence questions; if an individual answers in the affirmative to the occurrence question, then they proceed to the follow-up question to indicate how often the described situation happened (rarely, sometimes, or often) [25]. The recall period of the HFIAS is one month, and this questionnaire assesses food security at the household level [25]. The HFIAS categorizes food insecurity into four levels, and the degree of food insecurity increases with the number of affirmative answers [25]. Participants were categorized in one of the four categories of food insecurity: (i) food-secure, (ii) mildly food-insecure, (iii) moderately food-insecure, and (iv) severely food-insecure [25].

### 2.5. Data Analysis

Analyses were done using R version 4.3.1 and Statistical Package for Social Science (SPSS) version 29. Descriptive statistics such as frequencies, percentages, and mean ± standard deviation (SD) were calculated to summarize variables at baseline. To compare baseline characteristics between the intervention group and control group, the Wilcoxon rank sum test was used for non-normally distributed quantitative data, and independent t-tests were performed for normally distributed quantitative data. For categorical variables, a Chi-square test of independence was used. The significance level in this study was set at *p* < 0.05.

We evaluated the effect of the intervention on continuous outcomes using linear mixed effects models, with a random intercept implemented. For children’s growth outcomes, the random effect was the family code, as we had children from the same households enrolled in this study. The estimation procedure in the linear mixed effects models was maximum likelihood. The effect of the intervention on categorical outcomes was evaluated using the generalized estimating equations and the exchangeable working correlation structure. Factors adjusted in the liner mixed effects models and generalized estimating equations were chosen based on their potential effect on the outcome variable as documented in the literature [26,27,28].

For the generalized estimating equation analyses (MDD, ASFs, and prevalence of child undernutrition), multiple imputations were performed to resolve the missing data issues; see Appendix A Table A2 for the percentage of missing data for each study outcome at each assessment time point. The multiple imputations were performed using the mice package in R software, assuming that data were missing at random. Multiple imputations were not performed for the linear mixed effect model analyses, because previous results have shown very minimal to no difference in the results of LME models with or without minimal imputation of data, which was the case in our study [29]. Study investigators and outcome assessors were involved in the implementation of the intervention, and, therefore, were not blinded to participants’ group allocation.

## 3. Results

### 3.1. Participants Flow Chart

**Figure 3:**
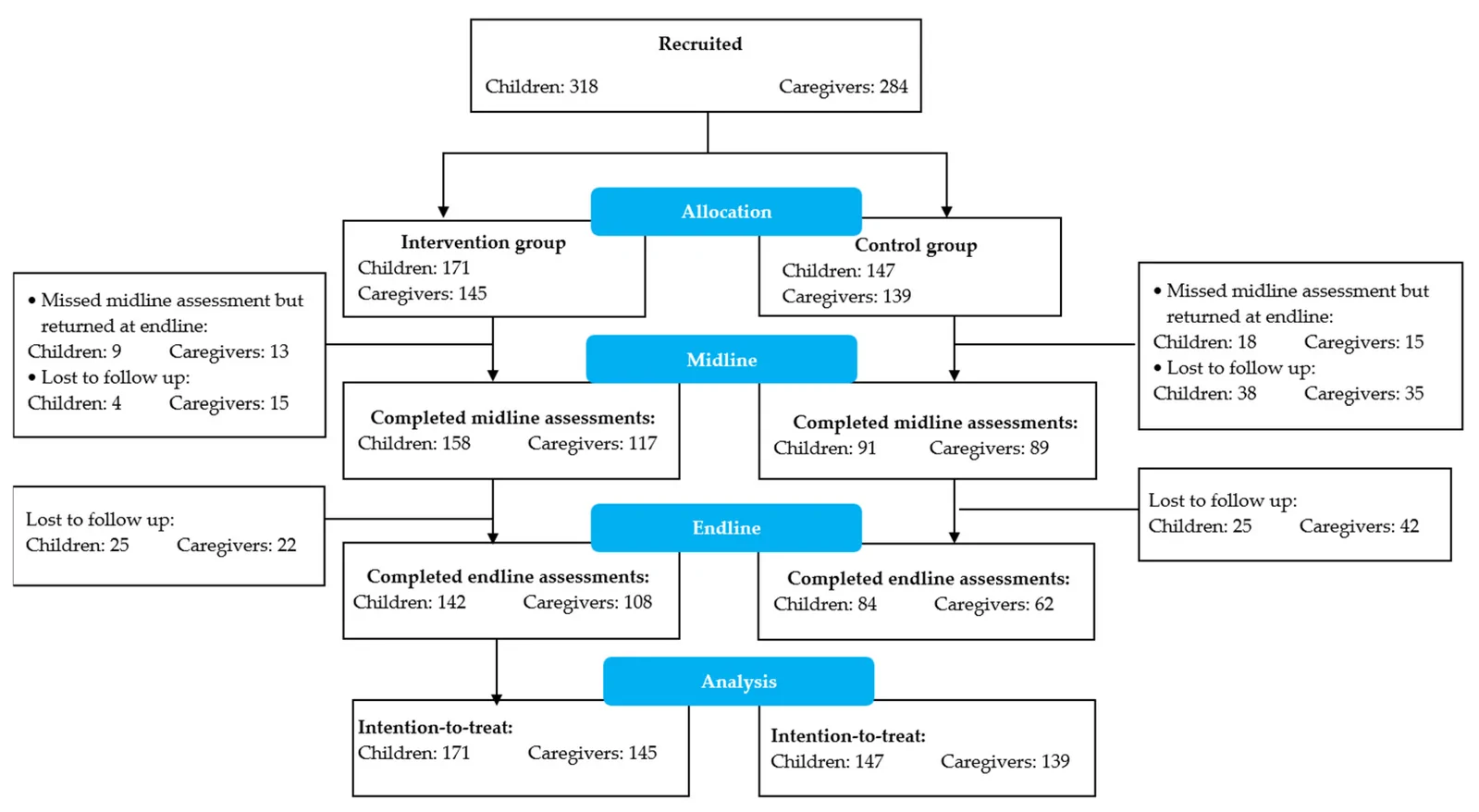
Flow diagram of recruitment and study participation.

A total of 318 children aged 6–59 months and 284 caregivers were recruited to participate in this study. The intervention group comprised 171 children and 145 caregivers, and the control group had 147 children and 139 caregivers. A total of 69 children and 78 caregivers missed the midline assessment. In total, 92 children and 114 caregivers were lost to follow-up (Figure 3). The demographic characteristics of children and their caregivers were comparable among those who missed midline assessments, those lost to follow-up, and those who attended both assessments.

### 3.2. Sociodemographic Characteristics and Food Security Status of Participants at Baseline

The mean age of participating children was 33.3 ± 16.8 months. Around 46.2% of children were female, while 53.8% were male. Most of the children’s caregivers (58%) were between 15 and 35 years old. The majority of caregivers (84.8%) were unable to read and write, and were housewives (66.8%). In addition, nearly all participants (99.6%) reported being moderately or severely food-insecure (Table 2).

### 3.3. Children’s Dietary Diversity

The mean dietary diversity score at baseline did not differ significantly between children in the intervention group (1.61 ± 0.93) and those in the control group (1.60 ± 0.83), *p* = 0.83. The adjusted linear mixed effects model indicated that, from baseline to the study endpoint, children in the intervention group had a significant increase in the dietary diversity scores compared to children in the control group (β = 0.98, 95% CI: 0.59, 1.37).

At baseline, only 1.8% and 2.9% of children met the minimum dietary diversity in the intervention group and control group, respectively, and this did not differ between the two groups (*p* = 0.45). Results from generalized estimating equations indicated that at the study endpoint, children in the intervention group were 8.40 times more likely to meet the minimum dietary diversity compared to children in the control group (Adjusted Odds Ratio (AOR): 8.40, 95% CI: 1.06, 66.78).

### 3.4. Consumption of Animal-Source Foods

At baseline, there was no significant difference in the proportion of children who consumed animal-source foods between the intervention group (31.0%) and the control group (28.8%) (*p* = 0.78). Following the intervention, children in the intervention group were 5.66 times more likely to consume animal-source foods compared to those in the control group (AOR: 5.66, 95% CI: 2.32, 13.77).

### 3.5. Children’s Meal Frequency

At baseline, mean meal frequency was comparable between children in the intervention group (2.1 ± 1.19) and those in the control group (2.0 ± 1.05), *p* = 0.18. The between-group comparison indicated that the increase in the meal frequency from baseline to the study endpoint was significantly higher in the intervention group compared to the control group (β = 0.77, 95% CI: 0.35, 1.20).

### 3.6. Effect of the Intervention on Child Nutrition Status

At baseline, children’s mean height was 92.1 ± 15.1 cm in the intervention group and 88.5 ± 12.8 cm in the control group, *p* = 0.04. Mean weight was 11.85 ± 3.30 kg in the intervention group and 11.27 ± 2.78 kg in the control group, *p* = 0.19. The mean WAZ was −1.14 ± 1.20 in the intervention group and −1.38 ± 1.34 in the control group, *p* = 0.01; mean WHZ was −1.58 ± 1.13 in the intervention group and −1.29 ± 1.07 in the control group, *p* = 0.005.

At baseline, 25.0% of children were affected by underweight (IG: 23.4% vs. CG: 26.5%, *p* = 0.52), 19.9% affected by stunting (IG: 12.9% vs. CG: 26.9%, *p* = 0.002), and 25.5% affected by wasting (IG: IG: 31.3% vs. CG 19.7%, *p* = 0.02).

We found no significant effect of the intervention on weight (β = 0.36, 95% CI: −0.09, 0.80), WAZ (β = 0.20, 95% CI: −0.06, 0.46), and WHZ (β = 0.17, 95% CI: −0.09, 0.42)—see Table 3. Similarly, there was no effect of the intervention on the prevalence of wasting (AOR: 0.76, 95% CI: 0.42, 1.38), and underweight (AOR: 0.82, 95% CI: 0.38, 1.77)—see Table 3.

## 4. Discussion

The aim of this study was to assess the effectiveness of an integrated community-based nutrition program, where caregivers received an intensive nutrition education intervention and their children consumed Harvest Lentil Vegetable Blend for eight weeks in Turkana, Kenya. The results of this study indicated that the intervention was effective in improving the quality and quantity of children’s diet. Additionally, this study observed a trend of greater improvement across key indicators of child nutrition status in the intervention group compared to the control group. However, none of these improvements were statistically significant.

This current study showed that the intervention significantly increased the frequency of meals among children in the intervention compared to those in the control group. It is noteworthy to mention that the dietary intake data for children were collected on days following a non-feeding day, hence the observed increase in meal frequency does not include the supplemental food provided to children. Our study aligns with a study conducted among caregivers of children aged 6–23 months in Bangladesh, where a 10-session culturally appropriate nutrition education intervention significantly increased the meal frequency of children in the intervention group compared to the control group [30]. Additionally, our study indicated that children in the intervention group were 8.40 times more likely to meet minimum dietary diversity compared to those in the control group. Our study compares favorably with that of [NO_PRINTED_FORM] [31], which found that a three-month nutrition education intervention was effective in increasing the proportion of children who met the minimum dietary diversity in a food-insecure rural community in Ethiopia.

Furthermore, the present study found that children in the intervention group had a sixfold higher likelihood of consuming animal-source foods compared to children in the control group at the study endpoint. This increase in the consumption of ASFs reflects the effectiveness of the nutrition educational intervention, which encouraged inclusion of ASFs in children’s diets which are readily available in this pastoralist community. A similar study conducted in rural Burkina Faso also found significantly higher consumption of animal-source foods among children in the intervention group compared to those in the control group, following a monthly nutrition education intervention implemented over a period of 10 months [32]. Additionally, similar results were reported in a study conducted in rural Ethiopia, where a 12-session nutrition education intervention significantly increased the proportion of children who consumed animal-source foods in the intervention group compared to the control group [33].

In this current study, we further assessed the effect of the integrated intervention on children’s nutrition status. Our study observed a positive trend in children’s nutrition outcomes. However, none of these trends reached statistical significance. These findings align with the MAHAY study in Madagascar, where children aged 6–18 months who received lipid-based nutrient supplementation for a year, coupled with intensive caregiver nutrition education, showed no significant differences in children’s Z-scores compared to children in the control group receiving standard care [34]. Furthermore, our study concurs with the IMIKA Program conducted in the Democratic Republic of Congo, where there were no significant differences between the growth indicators among children aged 6–12 months who received small quantities of lipid-based nutrient supplements on a monthly basis for six months while their caregivers received IYCF counseling for 2 years, and those in the control group [35].

This current study has some limitations. The majority of the data was self-reported, a method that could potentially introduce some recall bias. We also administered a single 24 h food recall per time point to assess children’s dietary intake, which does not capture daily variations in food consumption or seasonal variability in food. However, the pilot study found that children’s diet does not significantly change from day to day given the high levels of food insecurity in the study population [15]. There is also potential for social desirability bias, whereby participants may have reported behaviors they believed educators expected or wanted them to adopt. In this study we also had some dropouts, an issue that could have reduced our power to detect significant differences between the study arms. However, we used mixed effect models and imputed missing data to limit information bias [29,36]. It is also possible that the study duration (two months) was not as sufficient for us to detect small effects of the interventions on children’s nutrition status as we anticipated. Furthermore, given that individual randomization was unpractical in the study setting, we implemented a quasi-experimental study design. The lack of randomization and imbalance between participants in the study groups might have compromised the internal validity of the study. We minimized this risk by adjusting for relevant baseline characteristics in the study analyses.

## 5. Conclusions

This study demonstrated that an integrated nutrition intervention that coupled a theory-based nutrition education intervention with a blanket supplemental feeding program is effective in improving child-feeding practices in a pastoralist community in Kenya. The success of the intervention can be attributed to the systematic and comprehensive intervention design, where we used evidence-based practices such as behavior change theory, tailoring the intervention to the local context, incorporating health literacy principles, and providing sufficient dosages of the intervention content. Therefore, public health nutrition workforces working with similar populations should leverage these evidence-based practices when designing and implementing their nutrition education interventions. Although there was no significant effect of the intervention on children’s growth outcomes, the findings of this study highlight the potential of Harvest Lentil Vegetable Blend when combined with nutrition education intervention to improve children’s growth outcomes. Future studies should aim to provide more evidence of the effectiveness of Harvest Lentil Vegetable Blend when consumed for a longer time.

## Figures and Tables

**Table 1:** Description of the nutrition education modules.

Week, Module and Objective	Lesson Title and Objective(s)	Content	Activities and Used Visuals	TPB Construct
**Weeks 1 and 2** **Module 1:** Proper Breastfeeding Practices	The recommended breastfeeding practices. **Objectives:** At the end of the lesson, participants will be able to: Describe the benefits of breastfeeding a child during their first two years of life. Describe the benefits of breastfeeding to a mother. Describe the contents of breastmilk that make it superior to other types of milk.	Importance of breastfeeding to the child and the mother. Breastfeeding schedule during the first 6 months. What happens when a child receives mixed feeding before 6 months of age? Continued breastfeeding. Breastfeeding during sickness.	**Activity:** Discuss ways in which cultural norms in Turkana align and contrast with the recommended breastfeeding practices. **Used visual:** Breastfeeding during the first six months.	Knowledge; Attitudes; Subjective norms.
	Ensuring that your baby is getting enough breastmilk. **Objective:** At the end of the lesson, participants will be able to: Identify strategies to ensure that the child is getting enough breastmilk.	Feed the baby every time the baby wants to feed, both day and night. How to recognize that my baby is hungry. How to recognize that my baby is satisfied. How can I know that my baby is getting enough milk? Importance of hand expression of breastmilk. How to prepare a container for expressed milk. How to express milk by hand. Storage of expressed milk. How to feed babies expressed milk.	**Used visual:** Breastfeeding during the first six months. Hand expression of breastmilk poster.	Knowledge; Attitudes; Self-efficacy.
**Weeks 3 and 4** **Module 2:** Feeding My Baby a Healthy Diet	What food should I give to my baby? **Objectives:** At the end of the lesson, participants will be able to: Understand various nutrients and their benefits. Describe the recommended complementary feeding practices. Describe the consequences of poor complementary feeding practices	Basic principles of nutrition. The eight food groups your child should be feeding from. Tips on how to make sure that your baby’s meal contains various nutrients. How often should you feed your baby? How much should I feed my baby? What happens to my child when they are not fed properly?	**Activity:** How to feed children healthy meals with available resources? **Used visuals:** Food Groups for Children. Child Malnutrition poster.	Knowledge; Attitudes; Self-efficacy.
	Feeding children when they are sick. **Objective:** At the end of the lesson, participants will be able to: Describe how to feed children when they are sick.	Feeding and caring for sick children.	**Used visuals:** Food Groups for Children poster. Child Malnutrition poster.	Knowledge; Attitudes; Self-efficacy.
	Food selection and preparation. **Objective:** At the end of the lesson, participants will have increased confidence in choosing and preparing nutritious meals for their children.	Food selection; Food preparation.	**Used visual:** Food Groups for Children.	Self-efficacy.
**Weeks 5 and 6** **Module 3**: Food Safety, Water, Sanitation, and Hygiene	Food and water safety. **Objective:** At the end of the lesson, participants will be able to: Describe practical ways to ensure safe food preparation and handling. Know how to source and store potable drinking water.	Handling, preparing, and storing water in a clean and safe place. Handling, preparing, and storing food in a clean and safe place.	**Activity:** Ask participants to provide at least two tips for each of the main steps to keep food safe. Ask participants to discuss barriers to drinking safe water. **Used visual:** How to safely treat drinking water.	Knowledge; Attitudes; Self-efficacy.
	Hygiene **Objective:** At the end of the lesson, participants will be able to: Understand how to maintain good hygiene practices. Describe the advantages of using latrines when defecating.	Hygiene around the household. Personal hygiene.	**Activity:** How can we prevent open defecation in our community? Demonstration of proper ways to wash hands. **Used visuals:** Critical times to wash hands.	Attitudes; Subjective norms; Self-efficacy; Intentions.
**Weeks 7 and 8** **Module 4: Recap**	Recap on breastfeeding. **Objective:** At the end of the lesson, participants will: Increase their confidence in proper breastfeeding practices. Increase their intent to implement proper breastfeeding practices.	Breastfeeding during the first 6 months. Breastfeeding after the first 6 months.	**Activity:** Discuss and address the challenges mothers face when practicing exclusive breastfeeding and continued breastfeeding. **Used visuals:** Breastfeeding during the first six months. Hand expression of breastmilk.	Subjective norms; Self-efficacy; Intentions.
	Recap on feeding my baby a healthy diet. **Objective:** At the end of the lesson, participants will: Increase their confidence in feeding children healthy diets. Increase their intent to feed children healthy diets.	How to feed my baby at 6 months and older. Tips on how to make sure that your baby’s meal contains various nutrients. Feed your child frequently in a day.	**Activity:** Discuss and address the challenges mothers face when adopting proper complementary feeding practices. Ask for volunteers to name strategies they will adopt to make sure that their children receive sufficient and diverse food. **Used visuals:** Food Groups for Children; Child Malnutrition; Ensure food variety for your child.	Subjective norms; Self-efficacy; Intentions.
	Recap on food safety, water, sanitation, and hygiene. **Objective:** At the end of the lesson, participants will: Increase their confidence in implementing proper WASH practices for children and households. Increase their intent to implement proper WASH practices for children and households.	Food safety: four main steps to ensure that your food is safe (clean, separate, cook, and store). Treatment of drinking water: source, boil/chlorinate, and store. Washing hands. Use latrines when defecating.	**Activity:** **Part one:** Brainstorm the strategies and actionable goals to stop poor WASH practices in their communities. Ask for volunteers to name strategies they will adopt to make sure that their drinking water is safe and clean. **Used visuals:** How to safely treat drinking water. Critical times to wash hands.	Subjective norms; Self-efficacy; Intentions.

**Table 2:** Sociodemographic characteristics of Turkana children and their caregivers in Kenya at baseline.

Baseline Characteristic	Intervention Group	Control Group	*p* ^a^
	*n* (%)	*n* (%)	
Children (n = 318)			
Gender			
Female	79 (46.2)	68 (46.3)	0.992
Male	92 (53.8)	79 (53.7)	
Age in months, m ± SD	33.39 ± 16.70	33.12 ± 17.05	0.887
Age category			
6–12 months	35 (20.5)	26 (17.7)	0.530
13–36 months	75 (43.8)	65 (44.2)	0.949
37–59 months	61 (35.7)	56 (38.1)	0.655
Caregivers (*n* = 284)			
Caregivers’ age			
Adolescents	30 (20.7)	17 (12.3)	0.059
Young adults	75 (51.7)	88 (63.8)	0.040
Adults	40 (27.6)	33 (23.9)	0.480
Gender			
Female	136 (93.8)	137 (98.6)	0.076
Male	9 (6.2)	2 (1.4)	
Education level			
Unable to read and write	122 (84.1)	118 (85.5)	0.748
Can read and write and more	23 (15.9)	20 (14.5)	
Caregivers’ occupation			
Housewife	101 (69.7)	88 (63.8)	0.293
Casual laborers	31 (21.4)	25 (18.1)	0.491
Small-scale traders	10 (6.9)	14 (10.1)	0.327
Formal employment	3 (2.1)	9 (6.5)	0.079
Farmers	0 (0)	2 (1.4)	0.237
Own livestock			
Yes	95 (65.5)	71 (51.4)	0.016
No	50 (34.5)	67 (48.6)	
Livestock count, m ± SD	4.88 ± 5.95	3.48 ± 9.79	0.002
Food security status			
Food-secure	0 (0)	1 (0.7)	0.983
Moderately or severely food-insecure	145 (100)	138 (99.3)	

Note. ^a^ *p*-values for group comparisons for categorical data were calculated using the Chi-square test of independence, while *p*-values for continuous outcomes were calculated using the Wilcoxon rank sum test. The proportion of caregivers who owned livestock and the livestock count was significantly higher in the intervention group compared to the control group.

**Table 3:** Effects of the intervention on child nutrition status of Turkana children in Kenya.

Indicator		Intervention Group	Control Group	Between-Group Comparison, Δ(T_2_ − T_0_) †	
	Timepoint	Mean (95% CI)	Mean (95% CI)	Mean difference (95% CI)	*p*-value
Weight (kg)	T_0_	11.76 (11.21, 12.32)	11.28 (10.80, 11.76)	0.36 (−0.09, 0.80)	0.117
	T_1_	11.95 (11.38, 12.52)	11.38 (10.85, 11.91)		
	T_2_	12.25 (11.67, 12.84)	11.48 (10.94, 12.02)		
	Δ(T_2_ − T_0_)	0.49 (0.01, 0.97)	0.20 (−0.22, 0.62)		
	*p*-value	0.047	0.355		
Weight-for-Age Z-scores	T_0_	−1.17 (−1.37, −0.98)	−1.39 (−1.62, −1.15)	0.20 (−0.06, 0.46)	0.133
	T_1_	−1.01 (−1.21, −0.81)	−1.23 (−1.49, −0.98)		
	T_2_	−0.80 (−1.00, −0.59)	−1.18 (−1.43, −0.93)		
	Δ(T_2_ − T_0_)	0.37 (0.19, 0.56)	0.20 (0.06, 0.34)		
	*p*-value	<0.001	0.005		
Weight-for-Height Z-scores	T_0_	−1.61 (−1.78, −1.44)	−1.32 (−1.51, −1.12)	0.17 (−0.09, 0.42)	0.196
	T_1_	−1.60 (−1.78, −1.43)	−1.30 (−1.51, −1.08)		
	T_2_	−1.42 (−1.60, −1.24)	−1.29 (−1.51, −1.07)		
	Δ(T_2_ − T_0_)	0.19 (0.01, 0.37)	0.03 (−0.14, 0.19)		
	*p*-value	0.037	0.750		

Notes: T0, T1, T2: baseline, midpoint, and endpoint, respectively; † linear mixed effects model adjusted for children’s age and gender, caregivers’ education level and age, and livestock count, a proxy of wealth in Turkana County. The effect of the intervention was assessed using linear mixed effect models.

## Data Availability

The data that support the findings of this study are available from the corresponding author upon reasonable request and subject to applicable ethical and data-sharing restrictions.

## Appendix A

### Appendix A.1

**Table A1:** Nutrient composition of Harvest Lentil Vegetable Blend.

Nutrient	Harvest Lentil Vegetable Blend
Serving Size	100 g
Energy (Kcal)	344
Fat (g)	0.9
Carbohydrate (g)	67
Fiber (g)	8.8
Protein (g)	18.3
Potassium (mg)	644
Magnesium (mg)	45
Phosphorus (mg)	241
Zinc (mg)	2.5
Calcium (mg)	57.4
Iron (mg)	5.5
Iodine (mcg)	300
Manganese (mg)	1.3
Thiamin (B1) (mg)	0.7
Riboflavin (B2) (mg)	0.2
Vitamin B6 (mg)	0.5
Vitamin B12 (mcg)	0
Folate (B9) (mcg)	371
Niacin (B3) (mg)	3.1
Ascorbic acid (Vit C) (mg)	5
Pantothenic acid (B5) (mg)	1.7
Vitamin A (mcg RAE)	206.7

### Appendix A.2

**Table A2:** Number and percentage of missing outcome data by assessment time point and study group.

Outcome	Baseline, n Missing/N Expected (%)		Midline, n Missing/N Expected (%)		Endline, N Missing/N Expected (%)	
	Intervention group	Control group	Intervention group	Control group	Intervention group	Control group
Dietary diversity scores	0/145 (0%)	0/139 (0%)	28/145 (19.3%)	50/139 (36.0%)	37/145 (25.5%)	77/139 (55.4%)
% of children who met the minimum dietary diversity	0/145 (0%)	0/139 (0%)	28/145 (19.3%)	50/139 (36.0%)	37/145 (25.5%)	77/139 (55.4%)
% of children who consumed animal-source foods	0/145 (0%)	0/139 (0%)	28/145 (19.3%)	50/139 (36.0%)	37/145 (25.5%)	77/139 (55.4%)
Meal frequency	0/145 (0%)	0/139 (0%)	28/145 (19.3%)	50/139 (36.0%)	37/145 (25.5%)	77/139 (55.4%)
Weight (kg)	0/171 (0%)	0/147 (0%)	13/171 (7.6%)	56/147 (38.1%)	29/171 (17.0%)	63/171 (42.9%)
Weight-for-Age Z-scores	0/171 (0%)	0/147 (0%)	13/171 (7.6%)	56/147 (38.1%)	29/171 (17.0%)	63/171 (42.9%)
Weight-for-Height Z-scores	0/171 (0%)	0/147 (0%)	14/171 (8.2%)	57/147 (38.8%)	33/171 (19.3%)	65/171 (44.2%)
Wasting	0/171 (0%)	0/147 (0%)	14/171 (8.2%)	57/147 (38.8%)	33/171 (19.3%)	65/171 (44.2%)
Underweight	0/171 (0%)	0/147 (0%)	13/171 (7.6%)	56/147 (38.1%)	29/171 (17.0%)	63/171 (42.9%)
